# Leveraging Artificial Intelligence and Radiomics for Improved Nasopharyngeal Carcinoma Prognostication

**DOI:** 10.1002/cam4.70706

**Published:** 2025-03-19

**Authors:** Nicholas Brian Shannon, Narayanan Gopalakrishna lyer, Melvin Lee Kiang Chua

**Affiliations:** ^1^ Division of Surgery and Surgical Oncology, Department of Head and Neck Surgery National Cancer Centre Singapore Singapore; ^2^ Division of Surgery and Surgical Oncology, Department of Head and Neck Surgery Singapore General Hospital Singapore; ^3^ School of Public Health Imperial College London UK; ^4^ SingHealth Duke‐NUS Oncology ACP Singapore; ^5^ Division of Radiation Oncology National Cancer Centre Singapore Singapore

**Keywords:** locoregional recurrence, machine learning, nasopharyngeal carcinoma, radiomics, risk stratification

## Abstract

**Introduction:**

Nasopharyngeal carcinoma (NPC) typically presents as advanced disease due to the lack of significant symptoms in the early stages. Accurate prognostication is therefore challenging as current methods based on anatomical staging often lack the granularity to differentiate between patients with differing prognoses. This study investigates the potential of radiomics to improve the prediction of locoregional recurrence (LRR) and overall survival in patients with NPC.

**Methods:**

Radiomic features were extracted from radiotherapy planning CT scans for 294 NPC patients divided into training (*n* = 147) and validation (*n* = 147) sets. A feature selection step utilising feature clustering and mutual information classifier to select six key radiomic features was employed to reduce redundancy and improve interpretability. Models were trained using clinical data, radiomic features, and these in combination to predict 2‐year LRR, with performance assessed on the left‐out independent validation set.

**Results:**

Combining radiomic features with clinical data resulted in the best performance for predicting 2‐year LRR (Area Under the Curve, AUC 0.76) compared to prediction using clinical or radiomic features alone (mean AUC 0.56 and 0.57, respectively). Risk stratification based on the combined model was significant for LRR‐free survival and overall survival (*p* < 0.01). Key radiomic features included tumour size, intensity distribution, overall textural patterns, and distribution of fine and coarse textured regions.

**Discussion:**

Radiomics holds promise for improving NPC risk stratification, potentially allowing for personalised treatment strategies. The most important radiomics feature, maximum 2D diameter, suggests a need to reconsider tumour size as a prognostic criterion despite its current exclusion from TNM staging. Larger prospective studies are needed to validate these findings.

## Graphical abstract

This study investigates the potential of radiomics to improve risk stratification for locoregional recurrence (LRR) in nasopharyngeal carcinoma (NPC) patients. By combining radiomic features with clinical data, the authors achieved improved prediction of LRR compared to using either alone. The most important radiomics feature was tumour size, suggesting a need to reconsider its role in NPC staging.

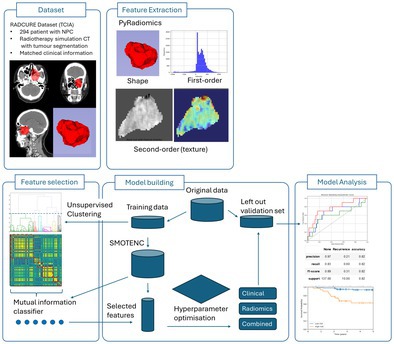

## 1 Introduction

Nasopharyngeal carcinoma (NPC) is endemic to specific geographic regions including East and South‐East Asia, and in these regions is often associated with the Epstein–Barr virus (EBV). Given its exquisite sensitivity to radiation and relative complexity of surgical access, radiotherapy is the primary treatment modality, with the inclusion of induction and/or concurrent systemic therapy based on tumour risk profile [1]. However, the frequent absence of early symptoms often leads to locally advanced disease at presentation [2] further complicating treatment and foretelling a less favourable prognosis [3].

The current standard of care for locoregionally advanced NPC is concurrent chemoradiotherapy. However, despite the success of this, locoregional recurrence (LRR) occurs in 10% of patients within 2–3 years [4]. Traditional staging systems based on anatomical information lack the granularity to differentiate patients with varying risks of recurrence. This is especially problematic given the many patients who present with locally advanced disease [2] and are therefore categorised as ‘high‐risk’ despite a significant proportion experiencing favourable outcomes [4]. Despite the promise of big data, analysis of large datasets has failed to yield significant improvements in stratification with clinical data alone [5]. Radiomics, the extraction of quantitative features from medical images such as CT scans, offers a promising solution. Unbiased identification of hidden patterns within medical images that correlate with a patient's clinical course can unmask tumour heterogeneity and microenvironmental characteristics not readily apparent to the expert radiologist's eye. This non‐invasive approach utilises scans already routinely performed for NPC patients, maximising the information gleaned from existing investigations. By extracting radiomics features with prognostic and predictive value, we can refine risk stratification.

This study aims to investigate the potential of radiomics to improve the prediction of LRR in patients with NPC. By harnessing additional information from standard imaging investigations, we hope to contribute to the ongoing efforts of refining patient stratification and allow more informed treatment decisions for NPC patients.

## 2 Methods

### 2.1 Data Source

The dataset used for this study consists of 355 NPC patients, as a subset of the RADCURE dataset which is publicly available from the Cancer Imaging Archive (TCIA) and is described in detail by Welch et al. [6, 7]. The entire dataset consists of 3346 head and neck cancer patients with computed tomography (CT) radiation therapy simulation images with corresponding gross‐tumour‐volume and organ‐at‐risk contours. From these, we included all patients with NPC that had a CT scan inclusive of primary tumour, segmentation of the tumour, and had clinical data available (*n* = 350). Staging was based on AJCC version 8.0.

**FIGURE 1:**
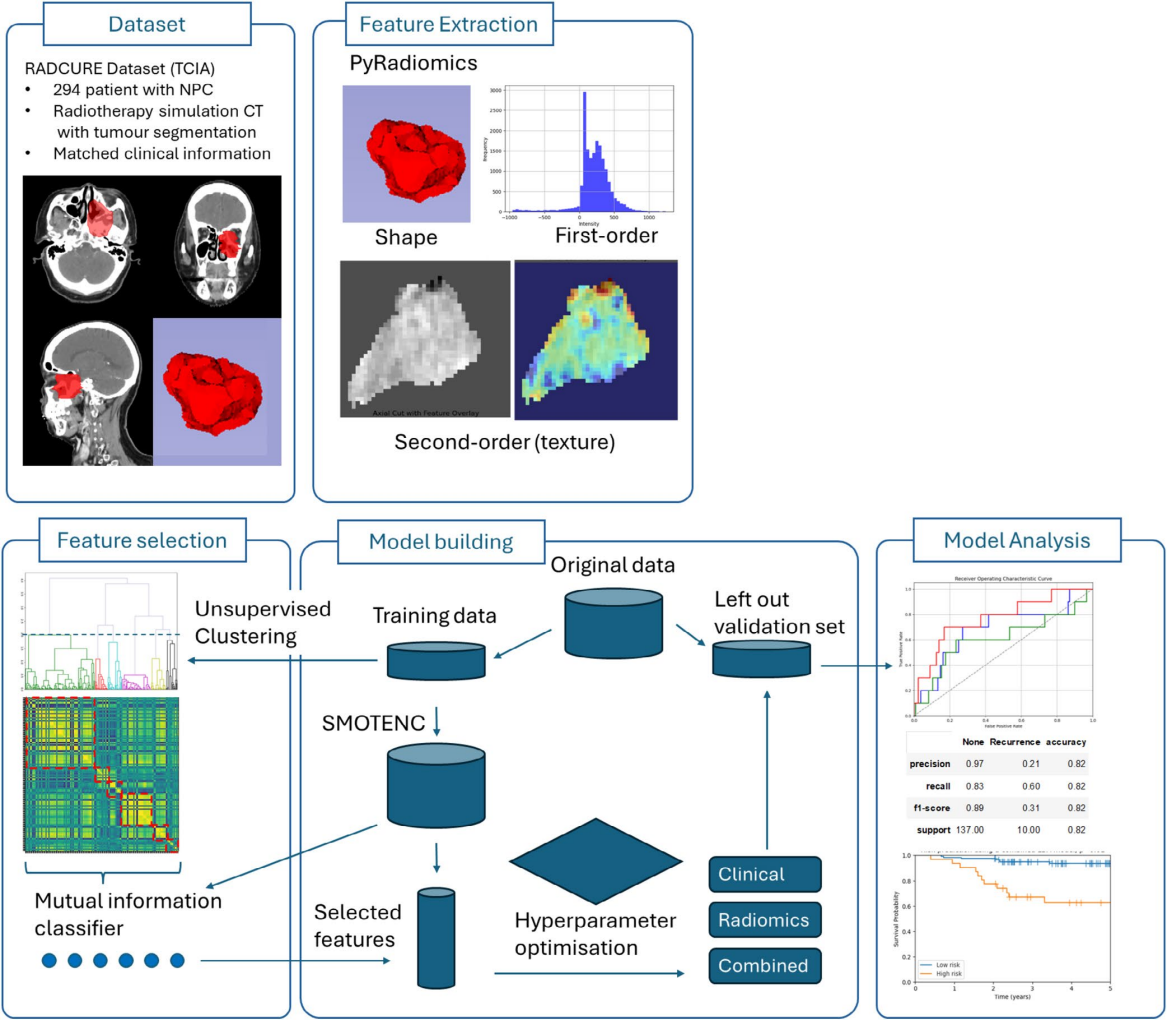
Outline of pipeline used for model development.

Patients were excluded if they had metastatic disease (*n* = 2) or less than 2 years of follow‐up if patients did not have LRR (*n* = 55). The dataset was randomly split into a training (*n* = 147) and validation (*n* = 147) dataset after feature extraction prior to any further processing. No transformation or oversampling of the independent validation set was performed, and the data in the validation set was not exposed until after model development to avoid any information leakage. Due to the class imbalance in our training data with rare LRR events, Synthetic Minority Oversampling Technique for nominal and continuous (SMOTENC [8]) from the Python package imblearn was employed on the training data to artificially generate synthetic samples for the minority class (LRR), balancing the class distribution and reducing overfitting to the majority class, giving a final training set of 272 samples (Figure 1).

### 2.2 Feature Extraction

Images were loaded and processed in python (version 3.12) using TorchIO (version 0.19), cropped to the segmentation mask representing gross‐tumour‐volume and two separate windowed images used for subsequent extraction, firstly with a level of 0 Hounsfield units (HU) and a width of 1000 HU covering from air to bone, and subsequently a narrower window with a level of 125 HU and a width of 350 HU to cover the expected range of mucosa and soft tissue in the head and neck region [9], thereby excluding regions consisting of the mask consisting only of air or bone. Aside from windowing, there was no additional preprocessing or filtering of images.

A total of 120 radiomics features were then extracted from the original images for each segmented gross‐tumour‐volume using PyRadiomics (version 3.1) with default parameter settings, including shape, first‐order and second‐order features.

Clinical categorical variables were one‐hot encoded, resulting in a binary representation for each category. To avoid redundancy and multicollinearity issues in the model, the first category of each one‐hot encoded variable was dropped.

### 2.3 Feature Selection

Given the inherent high dimensionality of radiomic data and the potential for redundancy between features, hierarchical clustering was used to identify potentially redundant features, aiming to reduce the feature space while retaining informative features for prediction. Spearman's rank correlation coefficient was calculated between all radiomic feature pairs in training data prior to SMOTENC oversampling and clustered using Ward's linkage. Feature clusters were defined by cutting the dendrogram using the SciPy (version 1.12.0) function fcluster at various distance thresholds, and a representative feature was chosen from each cluster using the scikit‐learn (version 1.4.1) function SelectKBest with the scoring function mutual information classification for features against LRR. A leave‐one‐out cross‐validation was used to evaluate the performance of reduced feature sets based on representative cluster features, and the elbow method was used to select the distance threshold that gave the best performance measured by the accuracy of a logistic regression model predicting LRR while reducing feature dimensionality.

### 2.4 Models

Prognostic models utilising radiomics features and/or clinical features to predict LRR were built using discriminative models (linear support vector machine [SVM], logistic regression, linear discriminant analysis [LDA], decision tree) and ensemble and boosted (random forest, AdaBoost, gradient boosting machine, XGBoost) algorithms. Hyperparameter optimisation was performed with a leave‐one‐out cross‐validation prior to fitting the model on the entire training dataset and then assessing performance in the validation dataset. We used the relevant implementation in the Python package Scikit‐learn (version 1.4.1) for model training and classification. For the purposes of discussion, patients predicted to have LRR were labelled as high risk and patients predicted to have no LRR were labelled as low risk.

## 3 Results

### 3.1 Dataset Characteristics

Of the 355 patients with NPC in the RADCURE dataset, 294 met inclusion and exclusion criteria, and of these, 21 had LRR within 2 years. The datasets were randomly divided into training (*n* = 147) and validation (*n* = 147), stratified by recurrence and without any significant differences in clinical features between the two datasets. Baseline characteristics of the 294 eligible patients are shown in Table 1.

**TABLE 1:** Characteristics of the training and validation set. ECOG PS = Eastern Cooperative Oncology Group performance status.

*n*	Overall	Training	Validation	*p*
	294	147	147	
Age, median [Q1, Q3]	52.1 [43.8,61.4]	49.8 [42.5,59.3]	53.6 [44.2,62.3]	0.142
Length of follow‐up (years), median [Q1, Q3]	5.7 [3.6,8.4]	6.2 [3.3,8.3]	5.6 [3.6,8.4]	0.741
Sex, *n* (%)				
Female	85 (28.9)	48 (32.7)	37 (25.2)	0.198
Male	209 (71.1)	99 (67.3)	110 (74.8)	
Smoking status, *n* (%)				
Current	51 (17.3)	20 (13.6)	31 (21.1)	0.359
Ex‐smoker	69 (23.5)	36 (24.5)	33 (22.4)	
Non‐smoker	166 (56.5)	86 (58.5)	80 (54.4)	
Unknown	8 (2.7)	5 (3.4)	3 (2.0)	
ECOG PS, *n* (%)				
ECOG 0	216 (74.0)	106 (72.6)	110 (75.3)	0.862
ECOG 1	72 (24.7)	38 (26.0)	34 (23.3)	
ECOG 2–3	4 (1.4)	2 (1.4)	2 (1.4)	
Subsite, *n* (%)				
Lateral wall	47 (16.0)	21 (14.3)	26 (17.7)	0.830
Posterior wall	34 (11.6)	16 (10.9)	18 (12.2)	
Superior wall	52 (17.7)	27 (18.4)	25 (17.0)	
Unknown	161 (54.8)	83 (56.5)	78 (53.1)	
T, *n* (%)				
T1	87 (29.6)	41 (27.9)	46 (31.3)	0.815
T2	32 (10.9)	18 (12.2)	14 (9.5)	
T3	98 (33.3)	48 (32.7)	50 (34.0)	
T4	77 (26.2)	40 (27.2)	37 (25.2)	
N, *n* (%)				
N0	34 (11.6)	12 (8.2)	22 (15.0)	0.392
N1	78 (26.5)	41 (27.9)	37 (25.2)	
N2	142 (48.3)	71 (48.3)	71 (48.3)	
N3a	15 (5.1)	9 (6.1)	6 (4.1)	
N3b	25 (8.5)	14 (9.5)	11 (7.5)	
M, *n* (%)				
M0	294 (100.0)	147 (100.0)	147 (100.0)	1.000
Stage, *n* (%)				
I	11 (3.7)	5 (3.4)	6 (4.1)	0.858
II	38 (12.9)	18 (12.2)	20 (13.6)	
III	136 (46.3)	66 (44.9)	70 (47.6)	
IV	109 (37.1)	58 (39.5)	51 (34.7)	
Treatment modality, *n* (%)				
ChemoRT	262 (89.1)	132 (89.8)	130 (88.4)	0.851
RT alone	32 (10.9)	15 (10.2)	17 (11.6)	
Type of recurrence, *n* (%)				
Local	19 (6.5)	9 (6.1)	10 (6.8)	0.996
Locoregional	6 (2.0)	3 (2.0)	3 (2.0)	
None	257 (87.4)	129 (87.8)	128 (87.1)	
Regional	12 (4.1)	6 (4.1)	6 (4.1)	
2‐year locoregional recurrence, *n* (%)				
No	273 (92.9)	136 (92.5)	137 (93.2)	1.000
Yes	21 (7.1)	11 (7.5)	10 (6.8)	

### 3.2 Radiomics Features

**FIGURE 2:**
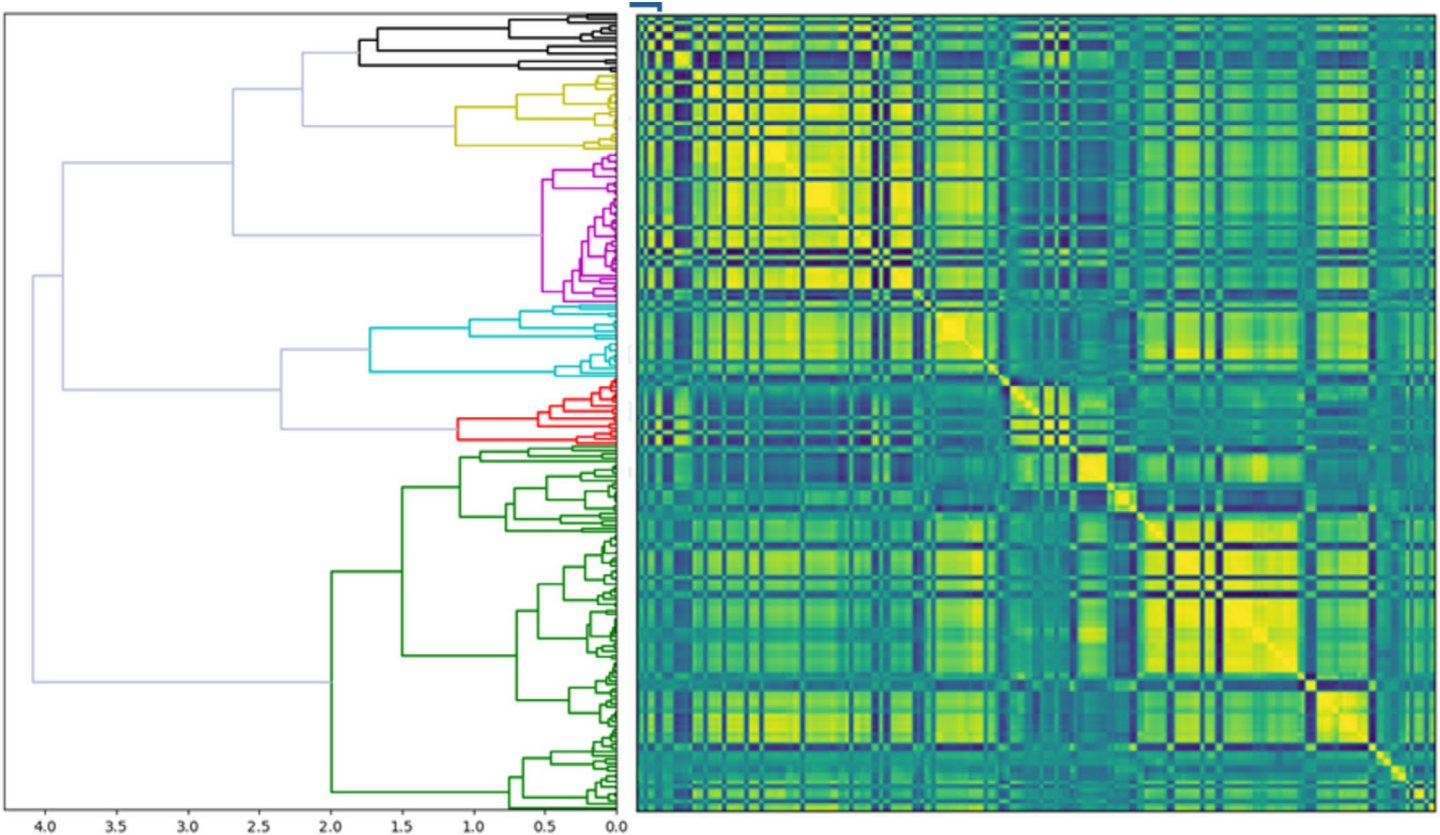
Unsupervised clustering of radiomics features utilized to identify six clusters (different colours on dendrogram) for selection of representative features.

The redundancy of extracted radiomics features was addressed using unsupervised clustering to group features with similar information across samples. Using a distance cut‐off of 2.0 and mutual information classifier, a representative radiomics feature was extracted for each of the six clusters (Figure 2, Table 2).

**TABLE 2:** Radiomics features selected for model training.

Shape	Maximum 2D diameter
First‐order	Median Interquartile range
Second‐order (texture)	Large dependence emphasis (LDE) Small area emphasis (SAE) Large area emphasis (LAE)

### 3.3 Model Performance

Models incorporating radiomics features achieved improved performance in predicting LRR compared to clinical features alone (Table 3). Although the overall performance of clinical and radiomics‐based models was comparable (mean AUC 0.56 range 0.5–0.63 vs. mean AUC 0.57 range 0.55–0.61), improved recall (0.2–0.4 to 0.6) without compromising overall performance was seen with discriminative linear models utilizing radiomics features. When clinical and radiomics features were combined, this improved performance across typical machine learning models and was able to reach good performance, where the best performing models utilised logistic regression (AUC 0.72) and LDA (AUC 0.76). The performance of ensemble and boosted machine learning models for all feature sets was poorer (AUC 0.5–0.61).

**TABLE 3:** Performance of prediction models in risk stratification for 2‐year locoregional recurrence.

Model	Clinical		Radiomics		Combined	
	Recall	AUC	Recall	AUC	Recall	AUC
Linear SVM	0.4	0.63	0.6	0.55	0.6	0.62
Logistic regression	0.2	0.55	0.6	0.60	0.6	0.72
Linear discriminant analysis	0.2	0.55	0.6	0.60	0.7	0.76
Decision tree	0.5	0.61	0.4	0.55	0	0.61
Random forest	0.3	0.61	0	0.56	0	0.51
AdaBoost	1	0.54	0.5	0.61	0.5	0.56
Gradient boosting	1	0.50	0	0.56	0	0.52
XGBoost	1	0.50	0	0.56	0.1	0.52

**FIGURE 3:**
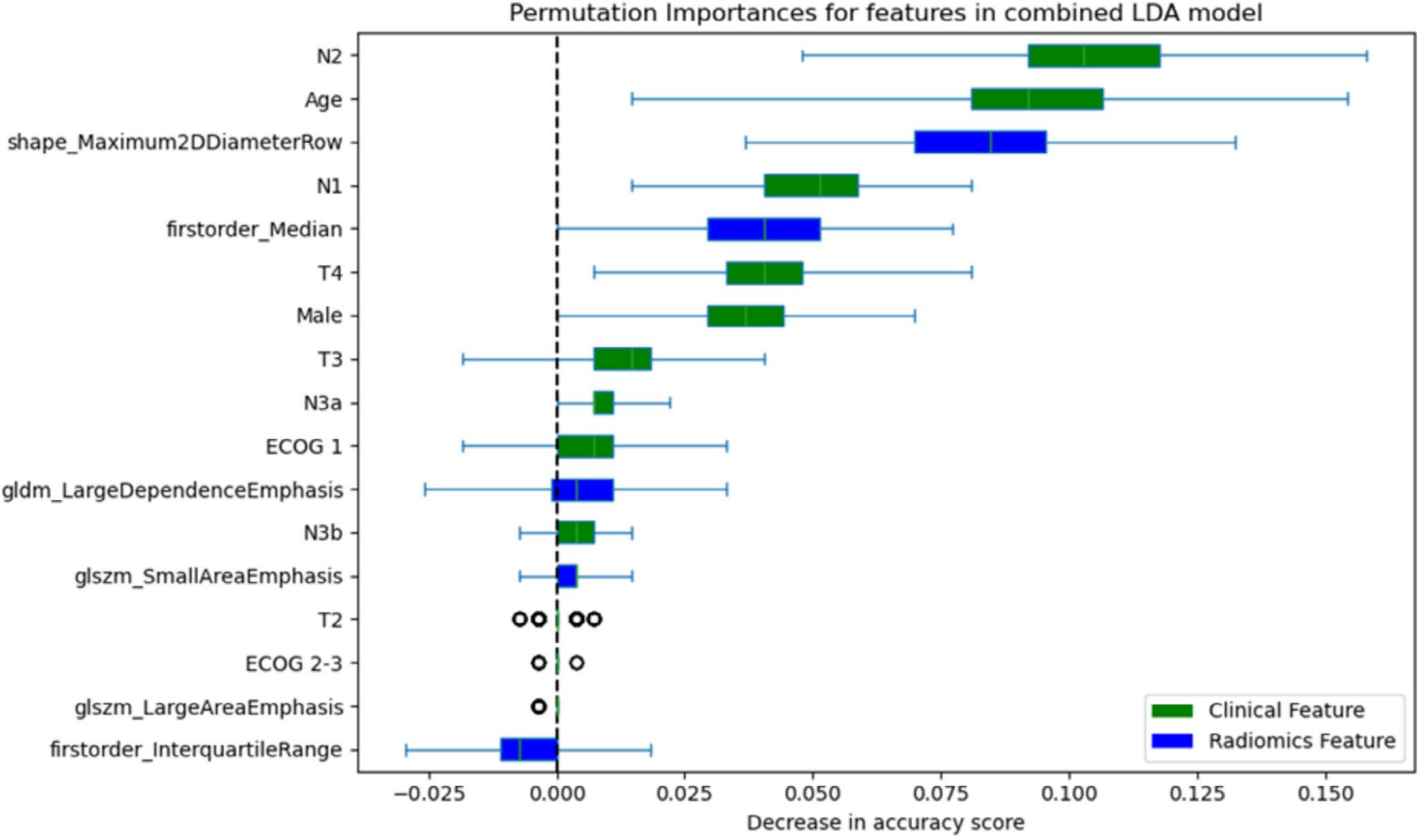
Permuted importance of features used in the combined linear discriminant analysis model.

Contribution of the features to the LDA model was assessed with permutation importance (Figure 3). Although the most important features reflected age and nodal status, radiomics features of maximum 2D diameter and first‐order median were important contributors to prediction accuracy.

**FIGURE 4:**
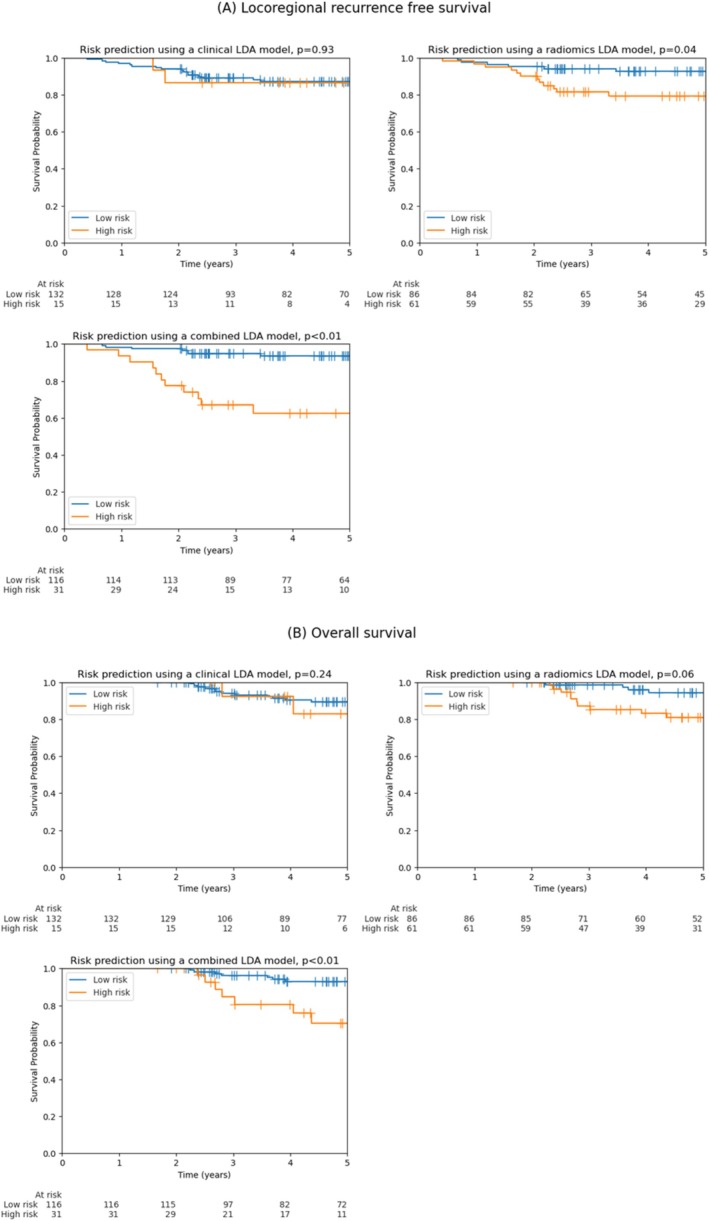
Locoregional recurrence‐free survival (A) and overall survival (B) by high and low‐risk groups predicted to have locoregional recurrence (high risk) or no locoregional recurrence (low risk) using clinical, radiomics and a combined model.

Patients predicted to have LRR (high‐risk group) by LDA models utilising radiomics had significantly worse LRR‐free survival (*p* = 0.04), and this was highly significant for a model combining both clinical and radiomics features (*p* < 0.01) (Figure 4A). This was also statistically significant for linear SVM and logistic regression models (Table S1). Similarly, patients predicted to have LRR had poorer overall survival, where this only reached significance for the combined model (*p* < 0.01) although a trend towards significance was observed for a model using only radiomics features (*p* = 0.06) (Figure 4B).

## 4 Discussion

Our study evaluated the performance of machine learning models utilising radiomics information for predicting LRR in NPC. By incorporating radiomic features alongside clinical data, clinicians can obtain a more accurate risk stratification, which has the potential to better personalise treatment strategies, potentially directing high‐risk patients towards more aggressive adjuvant therapies while avoiding unnecessary overtreatment or de‐escalating treatment in low‐risk patients. As radiotherapy is the primary treatment modality for NPC, the fact that patients will have undergone planning CT scans and will usually have tumour segmentation as part of radiotherapy planning means this group already meets the pre‐requisites for radiomics feature extraction, and prediction models could be readily implemented in the clinical setting.

Risk prediction based only on anatomical staging (tumour and nodal staging) and clinical features (age, sex and performance status) had poor discrimination for 2‐year LRR. Models using six radiomic features alone also had similar poor overall performance; however, they demonstrated improved recall and achieved significance for the prediction of LRR‐free survival due to later recurrences in high‐risk patients. Good risk stratification was only achieved by combining radiomic features alongside anatomical and clinical features, resulting in excellent risk stratification for LRR and overall survival.

Surprisingly, our analyses showed that the most important radiomics feature influencing locoregional failure was maximum 2D diameter, suggesting that perhaps overall tumour size is important for risk stratification. This is particularly interesting in NPC, where in contrast to many other cancers the primary tumour classification does not include size as a criterion but instead reports on anatomical location with regards to the nasopharynx and extension to adjacent areas or bony invasion. This is partly due to the infiltrative nature of NPC and difficulty in assessing size with conventional imaging approaches. However, the shift to the use of intensity‐modulated radiation therapy (IMRT) as the preferred radiotherapy technique and the development of computer‐aided segmentation in planning this offers easier delineation of tumour volume and assessment of size features. First‐order features of median and interquartile range capture the intensity distribution within the tumour; their inclusion might indicate that the heterogeneity of intensity levels within the tumour is informative. The biological basis for this is established by previous work in pancreatic ductal adenocarcinoma which has established a link between preoperative CT‐derived intensity and post‐operative histopathological cellularity [10]. The second‐order feature of large dependence emphasis reflects the prevalence of recurring intensity patterns within the tumour. High values indicate a larger dependence and more homogeneous textures, potentially reflecting a more organised and better‐differentiated tumour.

The feature has previously been associated with a complete pathological response in NPC after chemoradiotherapy [11] with higher values indicating a poorer response, in keeping with a known poorer response to radiotherapy in differentiated NPC [12]. A further potential link between texture and differentiation is highlighted by the importance of texture in differentiating between high‐grade and low‐grade gliomas [13]. Features of small area emphasis (SAE) and large area emphasis (LAE) describe the distribution of small and large area size zones, respectively. The balance between these two features could indicate a dominance of fine or coarse textured regions within the tumour, respectively. These might be related to specific biology or growth patterns with prognostic significance.

In NPC, CT imaging is often the primary imaging technique for radiation therapy planning due to its wide availability across centres, reduced scan time and lower expense than alternatives such as MRI. In addition, CT provides higher resolution of bone and vascular invasion, critical for anatomical staging. Although there has been work on the use of radiomics features extracted from CT scans, the previous studies are generally limited by small, underpowered sample sizes and short follow‐up times, which limit interpretation. Zeng et al. [14] explore the use of features extracted (*n* = 3668 extracted) from intra‐ and peri‐tumoral regions to predict disease‐free survival; however, their study is limited by relatively poor performance in a small testing set (*n* = 55) with short follow‐up and few survival events (*n* = 7), including a failure of Kaplan–Meier curves to split until a much later time point, despite the excellent performance in training data. Lin et al. [15] demonstrate progression risk stratification but are limited by a small dataset size (*n* = 66 training 33 test) and uncertain real‐world benefit given comparison to a clinical model consisting only of T and N stage, lacking established risk factors such as age and gender [16, 17]. Yan et al. [18] focus on locally advanced NPC and interestingly include biochemical markers such as LDH in their risk prediction model. However, similar limitations include a relatively small validation set (*n* = 93) and potential for overfitting given the large number of radiomics features assessed (1409 features extracted, and 20 remaining in their prediction model). Zhu et al. [19] similarly extract a large number of features (*n* = 1452 extract, *n* = 7 in the model) for a limited sample size (training set *n* = 109, validation set *n* = 47), and although predominantly CT‐based features, MRI‐fused images contribute to some of the shape features. Given the potential for high correlation among radiomic features, especially when derived from alternative image processing techniques, careful feature selection is crucial. Although the studies with a large number of features extracted employ LASSO regression for selection, ridge regression or an elastic net combination of the two may be preferred in such scenarios, as it is more effective in handling multicollinearity and leads to more stable coefficient estimates [20]. This may in part be responsible for the omission of tumour size from the features identified in these studies due to this being represented by multiple correlated features.

Our study is limited by its retrospective nature, which may have potential biases in patient selection that are not accounted for. Additionally, given the relatively low recurrence rate of NPC, the number of events for prediction validation is low; we have addressed this by ensuring a large enough validation dataset for robust performance assessment, and given a hazard ratio of 6.4 for LRR, our validation set is powered at approximately 100% in a post hoc power calculation. Finally, as a single‐centre study, the generalisability is weakened by a lack of independent external validation, including an assessment of the impact of differences in institutional scan protocols on model performance.

## 5 Conclusion

In conclusion, our findings corroborate previous research by demonstrating that radiomic features improve risk stratification in NPC patients. We have demonstrated this holds true using readily available planning CT scans and demonstrated superiority to standard clinical predictors. Notably, a key novel radiomics feature identified in this study is maximum 2D diameter, suggesting that tumour size might be an important omission from current TNM staging. Additionally, features reporting on heterogeneity and fine or coarse texture may capture additional information about tumour aggressiveness that is not captured by traditional clinical factors. Further validation of the radiomic prediction on a larger, prospective cohort is warranted. Additionally, while we identified significant radiomic features, their biological underpinnings remain unclear. Future work should focus on elucidating these links by comparing radiomic features with histopathological information and molecular profiling of tumour samples. This approach could shed light on NPC biology and potentially guide the development of novel therapeutic strategies.

## Author Contributions

**Nicholas Brian Shannon:** conceptualization (lead), data curation (lead), formal analysis (lead), investigation (lead), methodology (lead), visualization (lead), writing – original draft (lead), writing – review and editing (lead). **Narayanan Gopalakrishna lyer:** conceptualization (supporting), supervision (supporting), writing – review and editing (supporting). **Melvin Lee Kiang Chua:** conceptualization (supporting), methodology (supporting), supervision (equal), writing – review and editing (supporting).

## Conflicts of Interest

The authors declare no conflicts of interest.

## Supporting information


Appendix S1.

## Data Availability

The data used in this study are a subset of the RADCURE dataset publicly available on the Cancer Imaging Archive: https://doi.org/10.7937/J47W‐NM11.
