# Supplementary material for: Leveraging Artificial Intelligence and Radiomics for Improved Nasopharyngeal Carcinoma Prognostication
Source: Cancer Med. 2025 Mar 19;14(6):e70706. doi: 10.1002/cam4.70706 (PMC11921138; doi:10.1002/cam4.70706)
Supplement: Supplementary file 1 — Appendix S1. [file CAM4-14-e70706-s001.docx]

Supplementary

Feature selection


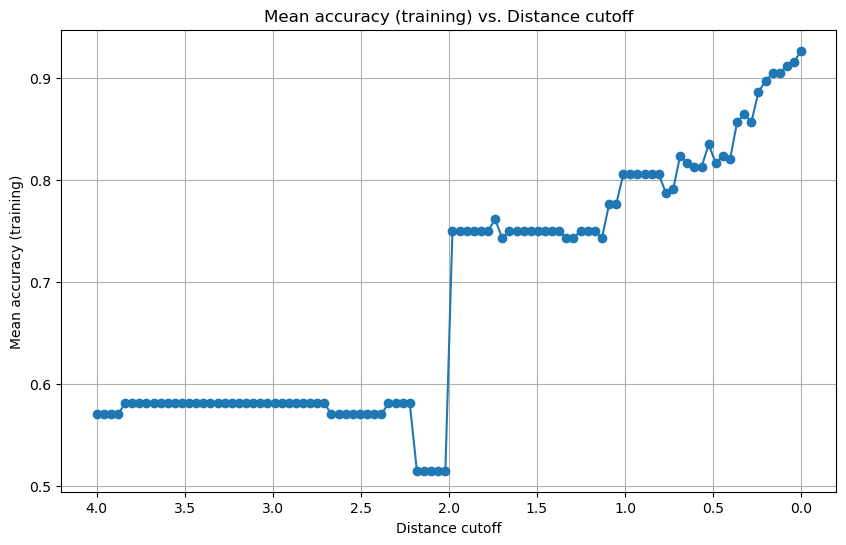


Figure S1. Accuracy of model in training dataset against distance cutoff used in feature selection.

Model performance

|  | Locoregional RFS | | | *Overall survival* | | |
| --- | --- | --- | --- | --- | --- | --- |
| Model | clinical | radiomics | combined | *clinical* | *radiomics* | *combined* |
| Linear SVM | 0.16 | 0.01 | 0.03 | <0.01 | 0.4 | 0.01 |
| Logistic Regression | 0.96 | 0.04 | <0.01 | 0.46 | 0.06 | <0.01 |
| LDA | 0.93 | 0.04 | <0.01 | 0.24 | 0.06 | <0.01 |
| Decision Tree | 0.39 | 0.55 | 0.09 | <0.01 | 0.78 | 0.86 |
| Random Forest | 0.21 | 0.51 | 0.51 | 0.01 | 0.47 | 0.54 |
| AdaBoost | 0.62 | 0.59 | 0.66 | 0.15 | 0.65 | 0.67 |
| Gradient Boosting | NaN | 0.91 | 0.39 | NaN | 0.93 | 0.41 |
| XGBoost | NaN | 0.38 | 0.92 | NaN | 0.2 | 0.49 |

Table S1. Significance (p-values) for logrank rest for model prediction of locoregional recurrence free survival (left) and overall survival (right)
